# PRE AND POST-OPERATIVE OTORHINOLARYNGOLOGY SURGERY CARE IN PATIENTS WITH GLYCOGEN STORAGE DISEASE TYPE 1

**DOI:** 10.1590/1984-0462/;2019;37;4;00005

**Published:** 2019-07-04

**Authors:** Adriana Maria Alves de Tommaso, Gabriel Hessel, Adriana Gut Riccetto, Graziela de Oliveira Semenzati, Reinaldo Jordão Gusmão

**Affiliations:** a Universidade Estadual de Campinas, São Paulo, SP, Brazil.

**Keywords:** Child, Glycogen storage disease, Hypoglycemia, Otolaryngology, Criança, Glicogenose, Hipoglicemia, Otolaringologia

## Abstract

**Objective:**

To discuss aspects of pre and post-operative otorhinolaryngology surgery in patients with glycogen storage disease type 1b.

**Case description:**

Description of three clinical cases with probable glycogen storage disease type 1b who underwent otorhinolaryngology surgery, showing the importance of multidisciplinary interaction to avoid episodes of hypoglycemia.

**Comments:**

Patients with glycogen storage disease type 1b present recurrent infections, including the otorhinolaryngology affections. When there is an indication for surgical treatment, the caloric intake should be carefully followed in order to prevent hypoglycemia. The way to ensure this is to perform the pre and postoperative period in the hospital ward. In the postoperative period, it is important to make a slow transition between the intravenous and oral routes and not suspend the infusion of glucose during the surgical procedure. The cases illustrate the need for the interaction of the otorhinolaryngologic surgeon with the anesthesiologist, the pediatrician and the gastro-pediatrician in the management of these patients, avoiding hypoglycemic episodes.

## RESUMO

**Objetivo:**

Discutir aspectos de pré e pós-operatório de cirurgia otorrinolaringológica em pacientes com glicogenose tipo 1b.

**Descrição do caso:**

Descrição de três casos clínicos com provável glicogenose tipo 1b, que se submeteram à cirurgia otorrinolaringológica, mostrando a importância da interação multidisciplinar para evitar os episódios de hipoglicemia.

**Comentários:**

Pacientes com glicogenose tipo 1b apresentam infecções de repetição, incluindo as otorrinolaringológicas. Quando há indicação de tratamento cirúrgico, deve-se observar a garantia de aporte calórico para evitar hipoglicemia. A maneira de fazer isso é efetuar o pré e pós-operatório em enfermaria, tomando-se o cuidado, no pós-operatório, de realizar uma transição lenta entre a via endovenosa e a via oral e de não suspender a infusão de glicose durante o procedimento cirúrgico. Os casos ilustram a necessidade da interação do otorrinolaringologista com o anestesista, o pediatra e o gastropediatra na condução desses pacientes para que não desenvolvam hipoglicemia.

## INTRODUCTION

Glycogen storage diseases or glycogenosis comprise a group of genetically determined diseases first described in 1928 and caused by abnormalities in enzymes that regulate the synthesis or degradation of glycogen.^1^ ^,^ ^2^ ^,^ ^3^ They cover 11 enzymatic deficiencies identified, classified numerically according to the specific enzymatic defect.^4^ The forms with a predominance of hepatic involvement are type 1, 3, 6 and 9, with an approximate frequency of one in 20,000 to 25,000 live births.^5^ ^,^ ^6^ ^,^ ^7^

Glycogen storage disease type 1 is the most common and severe type of glycogenosis. It is caused by a deficiency of the enzyme glucose-6-phosphatase (G-6-Pass) in the liver, kidney and intestine, thus the last phase of the glycogen degradation cascade into glucose does not happen.^6^ The most common clinical manifestations are: growth deficit, hepatomegaly, hypoglycemia, seizures, lactic acidosis, hyperuricemia, hypercholesterolemia and hypertriglyceridemia.

Types 1a and 1b are clinically similar, except for neutropenia and neutrophil dysfunction, which are type 1b characteristics. In these patients, neutrophils have reduced mobility and adherence, and defects in bactericidal activity and phagocytosis. Thus, patients have recurrent infections such as otitis, tonsillitis, pneumonia, cutaneous and urinary tract infections, and some infections require surgical treatment.

Upper respiratory tract infections, which are frequent in this disease, often cause an increase in obstruction of the lymphoid tissues (palatine and pharyngeal), indicating surgical removal to relieve symptoms and control infections. The management of these patients at the hospital requires greater care due to the risk of hypoglycaemia and in order to avoid damage to brain tissue.

Therefore, the purpose of this report is to discuss the management of patients with glycogen storage disease type 1b in otorhinolaryngological surgery regarding pre- and postoperative dietary aspects, in an attempt to minimize hypoglycemia episodes and their repercussion.

## DESCRIPTION OF THE CASES

Three cases of patients diagnosed with glycogen storage disease, probably type 1b, who underwent otorhinolaryngological surgery, are presented. The diagnosis of glycogen storage disease was based on the laboratory findings (hypoglycemia after three hours of fasting and increase of lactic acid, cholesterol, triglycerides and uric acid) and histological findings (pale hepatocytes, large fat vacuoles in the cytoplasm and cytoplasmic membrane resembling that of plant cells). Type 1b is likely due to the presence of significant neutropenia and recurrent infections. Deoxyribonucleic acid (DNA) was collected but not yet tested for the presence of mutations. The guidelines of gastropediatry in these cases that were referred for surgery were:

- Maintaining a 10% glucose infusion while the patient remains in fasting.
- Transitioning from intravenous hydration to slow oral diet according to the monitoring of blood glucose by means of reagent tapes every 6 hours and to the degree of patient acceptance.

These guidelines were not always followed by the doctors who treated the patients.

### Case 1

JMF, female, seven years old. Undergoing follow-up due to glycogen storage disease diagnosed at age three based on abnormalities found on laboratory tests and liver biopsy. During clinical evolution, she showed improvement of the metabolic alterations and normalization of the blood sugar levels. However, she had several episodes of tonsillitis, being referred for otorhinolaryngological evaluation. She was diagnosed with pseudomembranous pharyngotonsillitis in August 2011 and underwent clinical treatment. Face sinuses CT scan showed mucosal thickening of the left sphenoid sinus, undeveloped frontal sinuses, and other paranasal sinuses with normal aeration. In June 2012, she began taking granulokine because of neutropenia, and the diagnosis of glycogenosis type 1b was reached. In March 2013, she complained of repeated episodes of tonsillitis (about twice a month), and upon examination, hypertrophy of the tonsils was observed.

Surgery was performed on September 30, 2013. Fasting started at midnight, with infusion of glucose solution at a rate of 0.35 g/kg/hour. In the surgical center, 25-minute adenotonsillectomy was performed, and 100 mL of lactated Ringer’s was infused. Sent to the ward for recovery with infusion of 5% glucose solution. The glucose solution was changed to 10% while the patient did not accept a solid diet, and a transition was made according to oral acceptance and glycemic control via reagent strip. On the second postoperative day, the patient presented an episode of hypoglycemia, so the percentage of glucose in the liquid infusion was increased, and a slower intravenous/oral transition was planned. A cold liquid diet was maintained. Changed to a soft foods diet only on the seventh day. Discharged on the eighth postoperative day with good food intake, no hypoglycemia or other complications.

### Case 2

JVCGN, female, ten years old. Diagnosis of glycogen storage disease at 11 months of age. In the evolution, there was worsening neutropenia, reaching 260 neutrophils/mm^3^, indicating a probable case of glycogen storage disease type 1b. Throughout the follow-up, she developed recurrent respiratory infections, recurrent tonsillitis, and cervical adenitis. In August 2010, she attended the otorhinolaryngology outpatient clinic with a complaint of decreased auditory acuity. Upon examination, bilateral tympanic thickening and hyperaemia were present, bilaterally pale turbinates (2+/4+), and hypertrophic tonsils, with a diving component, as well as bilateral conductive hearing loss and bilateral type B curve immitanciometry in audiometry. Referred for bilateral tympanotomy with placement of a ventilation tube and adenotonsillectomy.

On February 7, 2011, adenotonsillectomy and tympanotomy were performed, with no secretory outflow, and the ventilation tube was not placed. During surgery, lactated Ringer’s was infused and blood glucose was measured by reagent strip, which showed an initial value of 34 mg/dL, which, after correction to 10% glucose solution, increased to 83 mg/dL. After returning from the surgical center, capillary glycemia was 49 mg/dL, and 10% glucose solution was reintroduced, as well as a cold liquid diet without lactose or sucrose. The transition happened quickly, as the patient showed good acceptance to the diet and was discharged on February 9, 2011.

In 2012, granulokine was started and, on October 8, 2013, a new procedure was performed for the placement of a ventilation tube in the left ear, under general anesthesia, without complications, with surgery time of one hour and 15 minutes. In the ward, she maintained metabolic control.

### Case 3

JATM, male, eight years old, undergoing follow-up due to glycogen storage disease from age one. He evolved with episodes of tonsillitis, furunculosis and adenoid hypertrophy. At that time, he was not on granulokine. He underwent adenotonsillectomy under general anesthesia on January 24, 2011. Surgery time was 30 minutes, and during the intra- and postoperative period, lactated Ringer’s was administered. In the immediate postoperative period, glycemia was at 50 mg/dL (per reactant strip), and 40 mL of 25% glucose solution were administered in bolus with good response. Slow transition from the intravenous route to oral diet because of pain. Discharged on the 10^th^ postoperative day, without episodes of hypoglycemia. He started granulokine in 2013 as advised by the pediatric immunology team.

## DISCUSSION

Glycogen storage disease type 1 is characterized by a deficiency of G-6-Pase, a key enzyme in glycogen metabolism. Since G-6-Pase is the leading supplier of glucose for circulation, its deficiency promotes an inability to maintain normal glucose levels during periods of fasting. Hypoglycaemia and its metabolic consequences are the main problem in these patients, and brain damage can occur due to recurrent hypoglycaemia.^8^ At an outpatient level, patients should consume small meals rich in complex carbohydrates every two to three hours during the day. In parallel, raw maize starch diluted in water is provided, interspersed with meals. Because they cannot be metabolized in G-6-Pase deficiency and because they contribute to an abnormal biochemistry, fruits, milk and dairy products are allowed in limited quantities as the child grows and depending on good metabolic control.

Given the low tolerance to fasting and the consequences of hypoglycemia, the routine of performing surgical procedures in an outpatient setting is not compatible with this type of patient. Through these reports, we advised patients to be hospitalized the day before surgery and administered a basal serum, with a glucose infusion of approximately 0.3-0.4 g/kg/h (equivalent to the administration of 10% glucose solution) during the preoperative fasting period.

When the patient is admitted to the surgical center, the anesthetist usually replaces the basal serum, which contains glucose, sodium and potassium, with lactated Ringer’s, which contains sodium chloride, potassium chloride, calcium chloride and sodium lactate, but not glucose, as occurred in all three cases. This can cause hypoglycemia, as verified in cases 2 and 3, fortunately without repercussion, since the patients were under surveillance during the hospitalization. Another possibility of developing hypoglycemia is upon returning to the ward, with infusion of lactated Ringer’s or 5% glucose solution, as happened in cases 1 and 2. There is still a risk of developing hypoglycemia when, in the postoperative period, a rapid transition is made from the parenteral route to the oral route for patient discharge.

There are few reports in the literature on the management of these patients when submitted to surgical procedures. Shenkman et al.^9^ indicated the anesthetic management of a 10-year-old patient with glycogen storage disease type 1b submitted to lithotripsy, in which mild hypoglycaemia was observed at the beginning of the surgical procedure. These authors recommend administering glucose at a dose of 4-8 mg/kg/min in the perioperative period, and intraoperatively, blood glucose levels should be carefully monitored.

Thus, based on these reports and the recommendations in the literature, the following protocol is proposed in the pre-, intra- and postoperative period in otorhinolaryngological surgery of patients with glycogen storage disease type 1b:

1. The preoperative period should be carried out in the ward to ensure adequate glucose supply by infusing fluid volume (10% glucose solution).^10^
2. Do not suspend glucose at the time of the surgical procedure, but ensure its administration at the same rate of the preoperative glucose infusion.
3. In the intraoperative period, monitor blood glucose levels through arterial blood gas test or reagent strip at the beginning of the procedure, and again every 30 minutes. If there is hyperglycemia, decrease the glucose infusion.
4. Pre- and post-operative blood glucose monitoring should be performed either through glucose dosing or preferably through reagent strips every 6 hours, or at shorter interval, if necessary.
5. Decreased glucose infusion in the postoperative period should only occur when the patient is ingesting 70 to 100% of the daily calorie requirement of a balanced diet. In addition, the percentage of infusion decrease should be low, a 25% figure every 12-24 hours. If, during the transition, blood glucose falls to levels below the normal range or the patient has hypoglycaemia, glucose infusion should be returned to the percentage of normal blood glucose levels.

These cases illustrate the need for the interaction between the otorhinolaryngologist and the anesthesiologist, with the pediatrician and with the gastropediatrist in the management of the dietary evolution of these patients, when they undergo otorhinolaryngological surgical procedures, so that they do not develop hypoglycemia with pertinent complications.
